# Supplementary figures and images for: An evaluation of the ecological niche of Orf virus (Poxviridae): Challenges of distinguishing broad niches from no niches
Source: PLoS One. 2024 Jan 18;19(1):e0293312. doi: 10.1371/journal.pone.0293312 (PMC10796068; doi:10.1371/journal.pone.0293312)

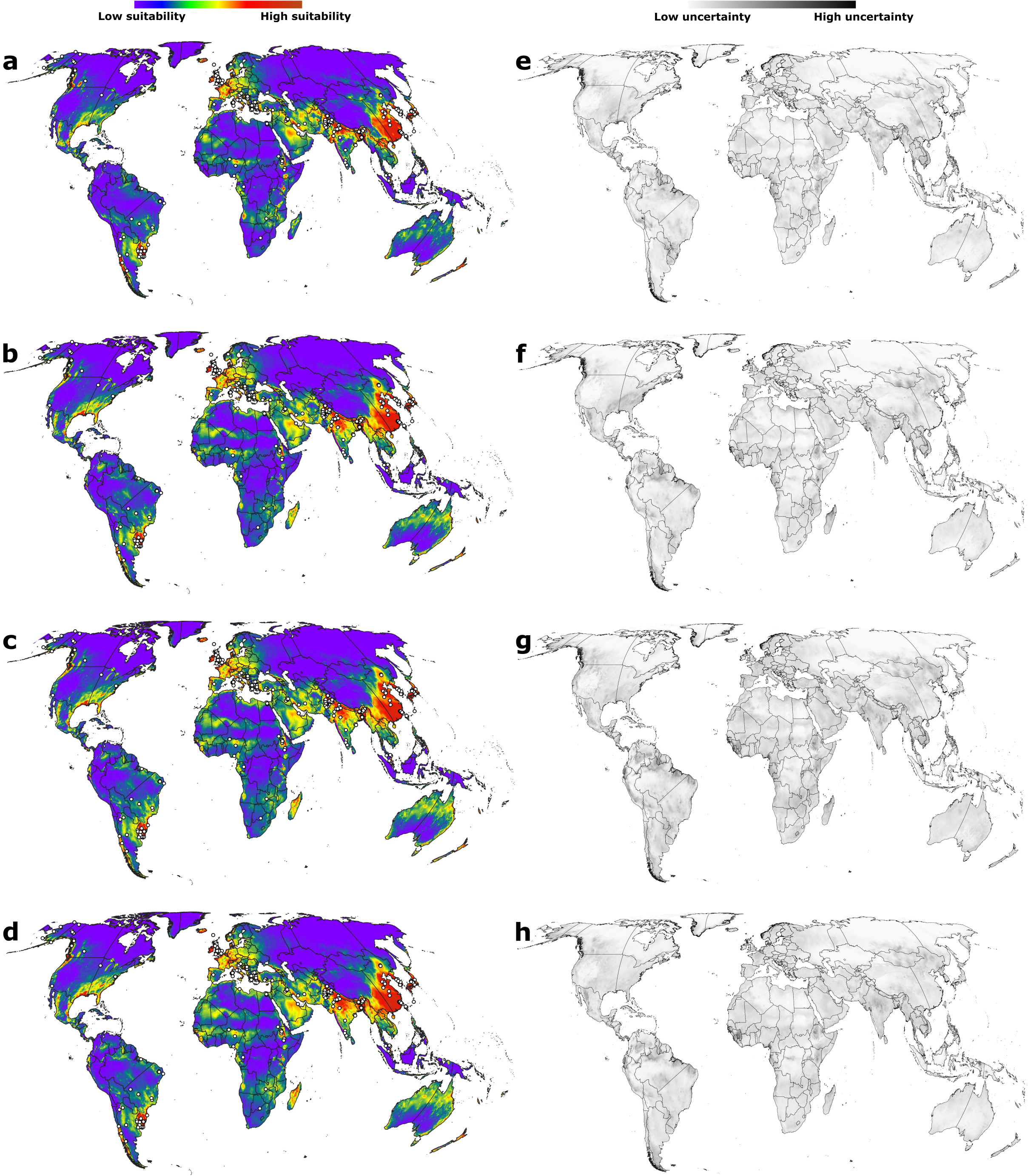

Supplement: S1 Fig — a-d) Median output of Maxent models displaying habitat suitability predictions. White circles indicate the known occurrence records, and e-h) corresponding uncertainties associated with those model predictions estimated as the range of suitability values among 10 model replicates. Free vector data were sourced from Natural Earth (naturalearthdata.com). (TIF) [file pone.0293312.s001.tif]
