# Supplementary material for: An evaluation of the ecological niche of Orf virus (Poxviridae): Challenges of distinguishing broad niches from no niches
Source: PLoS One. 2024 Jan 18;19(1):e0293312. doi: 10.1371/journal.pone.0293312 (PMC10796068; doi:10.1371/journal.pone.0293312)
Supplement: S1 Table — Details of occurrence records of Orf virus. (DOCX) [file pone.0293312.s002.docx]

| **Species** | **Latitude** | **Longitude** | **Country** | **Location** | **References** | **Remarks** |
| --- | --- | --- | --- | --- | --- | --- |
| ORF | 37.0600 | 21.6300 | Greece | Peloponnese | (Billinis et al., 2012) | Outbreak |
| ORF | 39.3900 | 21.9400 | Greece | Thessaly | (Billinis et al., 2012) | Outbreak |
| ORF | 12.7600 | 37.6300 | Ethiopia | Amba Giorgis | (Gelaye et al., 2016) | Outbreak |
| ORF | 12.5900 | 37.4600 | Ethiopia | Gondar zuria | (Gelaye et al., 2016) | Outbreak |
| ORF | 11.2600 | 37.4900 | Ethiopia | Adet | (Gelaye et al., 2016) | Outbreak |
| ORF | 8.7500 | 39.0200 | Ethiopia | Debre zeit | (Gelaye et al., 2016) | Outbreak |
| ORF | 7.8500 | 38.7100 | Ethiopia | Adami Tulu | (Gelaye et al., 2016) | Outbreak |
| ORF | 7.8500 | 38.7000 | Ethiopia | Adami Tulu | (Gelaye et al., 2016) | Outbreak |
| ORF | 37.6600 | 33.0600 | Turkey | Aksaray | (Şevik, 2017) | Outbreak |
| ORF | 37.8600 | 32.5100 | Turkey | Konya | (Şevik, 2017) | Outbreak |
| ORF | -37.0300 | -71.3500 | Argentina | Pichi-Neuquén | (Peralta et al., 2018) | Outbreak |
| ORF | -41.0500 | -70.4500 | Argentina | Comallo | (Peralta et al., 2018) | Outbreak |
| ORF | -25.3500 | -66.2800 | Argentina | La Aguadita | (Peralta et al., 2018) | Outbreak |
| ORF | -24.4000 | -63.6800 | Argentina | El Manantial | (Peralta et al., 2018) | Outbreak |
| ORF | -32.9100 | -65.4100 | Argentina | Concarán | (Peralta et al., 2018) | Outbreak |
| ORF | -41.1200 | -70.7100 | Argentina | Pilcaniyeu Town | (Peralta et al., 2015) | Outbreak |
| ORF | -15.5900 | -56.1000 | Brazil | Mato Grosso State | (Abrahão et al., 2009) | Outbreak |
| ORF | -20.6600 | -43.7800 | Brazil | Conselheiro Lafaiet | (Mazur et al., 2000) | Outbreak |
| ORF | -29.6800 | -53.8000 | Brazil | Santa Maria | (Mazur et al., 2000) | Outbreak |
| ORF | -8.0600 | -34.9200 | Brazil | Recife | (Mazur et al., 2000) | Outbreak |
| ORF | -1.1400 | -48.0800 | Brazil | Santo Antônio do Tauá | (Abrahão et al., 2012) | Outbreak |
| ORF | -7.0116 | -37.2688 | Brazil | Patos | (Nóbrega Jr et al., 2008) | Outbreak |
| ORF | -7.1699 | -37.5931 | Brazil | Catingueria | (Nóbrega Jr et al., 2008) | Outbreak |
| ORF | -6.8129 | -37.3798 | Brazil | São José de Espinharas | (Nóbrega Jr et al., 2008) | Outbreak |
| ORF | -7.1540 | -37.2992 | Brazil | São José do Bonfim | (Nóbrega Jr et al., 2008) | Outbreak |
| ORF | -19.7409 | -43.8261 | Brazil | Santa Luzia | (Nóbrega Jr et al., 2008) | Outbreak |
| ORF | -28.1287 | -48.9915 | Brazil | São Martinho | (Schmidt et al., 2013) | Outbreak |
| ORF | -31.2174 | -52.6695 | Brazil | Canguçú | (Schmidt et al., 2013) | Outbreak |
| ORF | -17.9096 | -51.7319 | Brazil | Jatai Municipality | (de Sant’Ana et al., 2013) | Outbreak |
| ORF | -34.2045 | -54.7614 | Uruguay | Aiguá | (da Costa et al., 2019) | Outbreak |
| ORF | -32.1474 | -56.1155 | Uruguay | Curtina | (Castro et al., 2019) | Outbreak |
| ORF | -30.5400 | -56.9500 | Uruguay | Artigas | (Olivero et al., 2018) | Outbreak |
| ORF | -31.2300 | -57.0500 | Uruguay | Salto | (Olivero et al., 2018) | Outbreak |
| ORF | -33.9300 | -54.0200 | Uruguay | Rocha | (Olivero et al., 2018) | Outbreak |
| ORF | -31.4600 | -55.2300 | Uruguay | Rivera | (Olivero et al., 2018) | Outbreak |
| ORF | -33.8600 | -55.0100 | Uruguay | Lavalleja | (Olivero et al., 2018) | Outbreak |
| ORF | -33.6900 | -55.9000 | Uruguay | Florida | (Olivero et al., 2018) | Outbreak |
| ORF | -32.9300 | -56.1100 | Uruguay | Durazno | (Olivero et al., 2018) | Outbreak |
| ORF | -34.1000 | -57.6700 | Uruguay | Colonia | (Olivero et al., 2018) | Outbreak |
| ORF | 62.3300 | 9.5000 | Norway | Dovre | (Vikøren et al., 2008) | Outbreak |
| ORF | 70.0300 | -107.5600 | Canada | Victoria Island | (Tomaselli et al., 2016) | Case study |
| ORF | 23.4800 | 91.5000 | India | Tripura | (Venkatesan et al., 2018) | Outbreak |
| ORF | 23.8300 | 91.3200 | India | Tripura | (Venkatesan et al., 2018) | Outbreak |
| ORF | 23.9100 | 91.8500 | India | Tripura | (Venkatesan et al., 2018) | Outbreak |
| ORF | 23.9200 | 91.8000 | India | Tripura | (Venkatesan et al., 2018) | Outbreak |
| ORF | 23.9300 | 91.8500 | India | Tripura | (Venkatesan et al., 2018) | Outbreak |
| ORF | 23.1600 | 91.4700 | India | Tripura | (Venkatesan et al., 2018) | Outbreak |
| ORF | 23.5600 | 91.5100 | India | Tripura | (Venkatesan et al., 2018) | Outbreak |
| ORF | 20.1300 | 85.4700 | India | Odisha | (Sahu et al., 2019) | Outbreak |
| ORF | 19.8000 | 85.8200 | India | Odisha | (Sahu et al., 2019) | Outbreak |
| ORF | 29.4700 | 79.6500 | India | Central Himalaya | (Mondal et al., 2006) | Outbreak |
| ORF | 27.1200 | 93.9300 | India | Laluk, Assam | (Bora et al., 2015) | Outbreak |
| ORF | 26.0527 | 91.8706 | India | Byrnihat, Assam | (Bora et al., 2015) | Outbreak |
| ORF | 26.1239 | 91.8205 | India | Khanapara, Assam | (Bora et al., 2015) | Outbreak |
| ORF | 27.1300 | 94.7400 | India | Demow, Assam | (Bora et al., 2015) | Outbreak |
| ORF | 26.1598 | 93.4053 | India | Karbi Anglong Assam | (Bora et al., 2015) | Outbreak |
| ORF | 26.2500 | 91.5200 | India | Hajo, Assam | (Bora et al., 2015) | Outbreak |
| ORF | 27.3300 | 77.7700 | India | Makhdum | (Kumar et al., 2014) | Outbreak |
| ORF | 10.2200 | 77.3500 | India | Tamil Nadu | (Nagarajan et al., 2019) | Outbreak |
| ORF | 24.2993 | 73.5316 | India | Chokri, Rajasthan | (Maan et al., 2014) | Outbreak |
| ORF | 29.4692 | 79.6501 | India | Mukteshwar | (Hosamani et al., 2007) | Outbreak |
| ORF | 30.8100 | 75.8200 | India | Ludhiana District | (Yogisharadhya et al., 2012) | Outbreak |
| ORF | 17.3963 | 78.4673 | India | Hyderabad | (Karki et al., 2020) | Outbreak |
| ORF | 28.4065 | 79.4407 | India | Bareilly City | (Sharma et al., 2016) | Case study |
| ORF | 29.6000 | 114.5200 | China | Hubei province | (Zhang et al., 2010) | Outbreak |
| ORF | 31.7300 | 115.9200 | China | Luan | (Wang et al., 2019) | Outbreak |
| ORF | 26.5600 | 118.2600 | China | Nanping | (Chi et al., 2017) | Outbreak |
| ORF | 44.9200 | 124.5200 | China | Nongan | (Li et al., 2012) | Outbreak |
| ORF | 33.5459 | 113.6678 | China | Wuyang | (Chen et al., 2017) | Outbreak |
| ORF | 26.1300 | 119.1200 | China | Farm | (Chi et al., 2013) | Outbreak |
| ORF | 25.4000 | 119.5200 | China | Farm | (Chi et al., 2013) | Outbreak |
| ORF | 25.9100 | 119.2800 | China | Farm | (Chi et al., 2013) | Outbreak |
| ORF | 26.4400 | 118.4300 | China | Farm | (Chi et al., 2013) | Outbreak |
| ORF | 26.4400 | 118.4200 | China | Farm | (Chi et al., 2013) | Outbreak |
| ORF | 5.2821 | 103.0806 | Pen. Malaya | Kuala Terengganu | (Bala et al., 2019) | Herd health programme |
| ORF | 5.0600 | 103.2100 | Pen. Malaya | Marang | (Bala et al., 2019) | Herd health programme |
| ORF | 5.4865 | 102.7629 | Pen. Malaya | Setiu | (Bala et al., 2019) | Herd health programme |
| ORF | 2.7300 | 101.9400 | Malaysia | GBIF | GBIF | Not known |
| ORF | 23.8434 | 120.9815 | Taiwan | Nantou | (Chan et al., 2007) | Outbreak |
| ORF | 24.2381 | 120.8932 | Taiwan | Taichung | (Chan et al., 2009) | Outbreak |
| ORF | 14.4000 | 35.8000 | Sudan | Gedarif | (Khalafalla et al., 2020) | Outbreak |
| ORF | 5.0082 | 7.9167 | Nigeria | Uyo | (Adedeji et al., 2017) | Outbreak |
| ORF | 13.0100 | 5.2500 | Nigeria | Sokoto | (Lawal, 2020) | Outbreak |
| ORF | 44.8600 | 14.4000 | Croatia | Island of Cres | (Lojkic et al., 2010) | Outbreak |
| ORF | 45.6600 | 15.9600 | Croatia | Turopolje region | (Lojkic et al., 2010) | Outbreak |
| ORF | 69.1100 | -105.3000 | Canada | Victoria Island | (Tomaselli et al., 2022) | Case study |
| ORF | 62.0695 | -6.8913 | Faroe Islands | Faroe Islands | (Villadsen and Zachariae, 2008) | Case study |
| ORF | 52.0571 | -2.7220 | England | Hereford | (Paiba et al., 1999) | Case study |
| ORF | 52.6323 | 1.2823 | England | Norwich | (Paiba et al., 1999) | Case study |
| ORF | 53.8121 | -2.6977 | England | Preston | (Paiba et al., 1999) | Case study |
| ORF | 36.2700 | 136.6700 | Japan | Hakusan-shi | (Ito et al., 2009) | Case study |
| ORF | 35.3700 | 137.4200 | Japan | Ena | (Inoshima et al., 2010) | Outbreak |
| ORF | 37.4000 | 140.3000 | Japan | Koriyama | (Inoshima et al., 2010) | Outbreak |
| ORF | 36.1995 | 137.8165 | Japan | Matsumoto | (Inoshima et al., 2010) | Outbreak |
| ORF | 35.0300 | 137.2100 | Japan | Aichi | (Inoshima et al., 2010) | Outbreak |
| ORF | 36.7700 | 136.7700 | Japan | Ishikawa | (Inoshima et al., 2010) | Outbreak |
| ORF | 36.6400 | 137.2600 | Japan | Toyama | (Inoshima et al., 2010) | Outbreak |
| ORF | 36.0800 | 137.3300 | Japan | Forest area (Gifu) | (Suzuki et al., 1993);(Inoshima et al., 2002) | Case study |
| ORF | 26.4955 | 127.9595 | Japan | Okinawa | (Inoshima et al., 2002) | Outbreak |
| ORF | 36.4300 | 136.5400 | Japan | Nomi-shi | (Ito et al., 2009) | Outbreak |
| ORF | 32.7902 | 13.3361 | Libya | Tarabulus Municipality | (Azwai et al., 1995) | Outbreak |
| ORF | 39.0064 | -77.0192 | USA | Walter Reed Army Institute of Research | (Kinley et al., 2013) | Case study |
| ORF | 59.1232 | -135.5022 | USA | Haines | (Tryland et al., 2018) | Case study |
| ORF | 55.5600 | -131.3700 | USA | Ketchikan | (Tryland et al., 2018) | Case study |
| ORF | 58.4526 | -134.2331 | USA | Juneau | (Tryland et al., 2018) | Case study |
| ORF | 64.4900 | -164.7500 | USA | Safety Sound Nome | (Tryland et al., 2018) | Case study |
| ORF | 64.8319 | -147.6565 | USA | Fairbanks | (Zarnke et al., 1983) | Case study |
| ORF | 34.3300 | -118.2200 | USA | Los Angeles County | (Lo and Mathisen, 1996) | Case study |
| ORF | 44.7458 | -93.2010 | USA | Minnesota (Zoologicl garden) | (Guo et al., 2004) | Outbreak |
| ORF | 25.4400 | 50.9500 | Qatar | Leawaina | (Shehata et al., 2021) | Outbreak |
| ORF | 40.5700 | 8.4400 | Italy | Putifigari | (Coradduzza et al., 2021) | Outbreak |
| ORF | 40.7700 | 8.4100 | Italy | Sassari | (Coradduzza et al., 2021) | Outbreak |
| ORF | 41.0900 | 9.4100 | Italy | Arzachena | (Coradduzza et al., 2021) | Outbreak |
| ORF | 40.5575 | 8.8470 | Italy | Mores | (Coradduzza et al., 2021) | Outbreak |
| ORF | 39.2213 | 9.0960 | Italy | Cagliari | (Coradduzza et al., 2021) | Outbreak |
| ORF | 40.9000 | 9.1000 | Italy | Tempio Pausania | (Coradduzza et al., 2021) | Outbreak |
| ORF | 31.5800 | 44.5000 | Iraq | AL-Dagharah district | (Mansour et al., 2021) | Outbreak |
| ORF | 33.0200 | 45.8600 | Iraq | Badrah | (Hussain et al., 2022) | Outbreak |
| ORF | 32.1400 | 45.9400 | Iraq | Al Hayy | (Hussain et al., 2022) | Outbreak |
| ORF | 32.3600 | 45.4800 | Iraq | Al Numaniyah | (Hussain et al., 2022) | Outbreak |
| ORF | 29.6500 | 73.0600 | Pakistan | Bahawalnagar District | (Hussain et al., 2022) | Outbreak |
| ORF | 28.8100 | 71.7200 | Pakistan | Bahawalpur District | (Hussain et al., 2022) | Outbreak |
| ORF | 30.0700 | 70.2200 | Pakistan | Dera Ghazi Khan District | (Hussain et al., 2022) | Outbreak |
| ORF | 31.6500 | 71.4000 | Pakistan | Bhakkar District | (Hussain et al., 2022) | Outbreak |
| ORF | 31.3100 | 72.3400 | Pakistan | Jhang District | (Hussain et al., 2022) | Outbreak |
| ORF | 31.0500 | 74.1300 | Pakistan | Kasur District | (Hussain et al., 2022) | Outbreak |
| ORF | 32.1700 | 72.0700 | Pakistan | Khushab District | (Hussain et al., 2022) | Outbreak |
| ORF | 31.4600 | 74.3500 | Pakistan | Lahore District | (Hussain et al., 2022) | Outbreak |
| ORF | 30.9800 | 71.2400 | Pakistan | Layyah | (Hussain et al., 2022) | Outbreak |
| ORF | 29.6500 | 71.7200 | Pakistan | Lodhran | (Hussain et al., 2022) | Outbreak |
| ORF | 29.9100 | 71.4000 | Pakistan | Multan | (Hussain et al., 2022) | Outbreak |
| ORF | 30.0300 | 71.0200 | Pakistan | Muzaffargarh | (Hussain et al., 2022) | Outbreak |
| ORF | 29.1400 | 70.1500 | Pakistan | Rajan Pur | (Hussain et al., 2022) | Outbreak |
| ORF | 28.4000 | 70.5100 | Pakistan | Rahim Yar Khan | (Hussain et al., 2022) | Outbreak |
| ORF | 30.2700 | 57.0499 | Iran | Kerman | (Oryan et al., 2017) | Outbreak |
| ORF | 29.6228 | 52.5152 | Iran | Shiraz | (Oryan et al., 2017) | Outbreak |
| ORF | 12.1239 | 37.7808 | Ethiopia | Addis Zemen | (Tedla et al., 2018) | Outbreak |
| ORF | 29.8999 | 31.2634 | Egypt | El-Hawamdeyya Township | (Mahmoud et al., 2010) | Outbreak |
| ORF | 52.4100 | -115.7500 | Canada | Ram Mountains | (L’Heureux et al., 1996) | Outbreak |
| ORF | -24.7747 | 25.8536 | Botswana | Gaborone | (Baipoledi et al., 2002) | Outbreak |
| ORF | -0.1585 | 13.7266 | Gabon | Tebe | (Maganga et al., 2016) | Outbreak |
| ORF | -33.5421 | -70.8469 | Chile | San Bernardo | (Flores et al., 2017) | Case study |
| ORF | -15.4262 | 28.3357 | Zambia | Lusaka city | (Simulundu et al., 2017) | Outbreak |
| ORF | 36.4647 | 115.8829 | China | Liaocheng | (Zhang et al., 2015) | Outbreak |
| ORF | 36.0000 | 117.0357 | China | Tai’an City | (Zhang et al., 2015) | Outbreak |
| ORF | 35.3809 | 116.6735 | China | Jining City | (Zhang et al., 2015) | Outbreak |
| ORF | 41.0123 | 24.4012 | Greece | Kavala | (Kottaridi et al., 2006) | Outbreak |
| ORF | 39.2600 | 26.2700 | Greece | Lesbos | (Kottaridi et al., 2006) | Outbreak |
| ORF | 39.3815 | 22.7450 | Greece | Velestino | (Kottaridi et al., 2006) | Outbreak |
| ORF | 37.4424 | 22.3948 | Greece | Arcadia | (Kottaridi et al., 2006) | Outbreak |
| ORF | 38.4022 | 26.0165 | Greece | Chios | (Kottaridi et al., 2006) | Outbreak |
| ORF | 41.1128 | 25.5046 | Greece | Rhodope | (Kottaridi et al., 2006) | Outbreak |
| ORF | 42.3950 | 13.4249 | Italy | L’Aquila | (Kottaridi et al., 2006) | Outbreak |
| ORF | 42.2536 | 13.9342 | Italy | Torre de’ Passeri | (Kottaridi et al., 2006) | Outbreak |
| ORF | 42.1868 | 14.2443 | Italy | Guardiagrele | (Kottaridi et al., 2006) | Outbreak |
| ORF | 42.3023 | 12.4382 | Italy | Civita Castellana | (Kottaridi et al., 2006) | Outbreak |
| ORF | 34.8683 | 126.9015 | South Korea | Jeonnam province | (Oem et al., 2013) | Outbreak |
| ORF | 37.7175 | 128.2810 | South Korea | Gangwon province | (Oem et al., 2013) | Outbreak |
| ORF | 37.4928 | 127.1850 | South Korea | Gyeonggi-do province | (Oem et al., 2013) | Outbreak |
| ORF | 36.3180 | 128.7462 | South Korea | Gyeongbuk province | (Oem et al., 2013) | Outbreak |
| ORF | 36.8000 | 127.7000 | South Korea | Chungbuk province | (Oem et al., 2009) | Outbreak |
| ORF | 30.2990 | 31.2777 | Egypt | Qalyubia | (Selim et al., 2016) | Outbreak |
| ORF | 34.1300 | 75.0300 | India | Dachigam | (Ahanger et al., 2018) | Outbreak |
| ORF | 34.1364 | 74.8804 | India | Nishat | (Ahanger et al., 2018) | Outbreak |
| ORF | 34.1800 | 74.8200 | India | Shuhama | (Ahanger et al., 2018) | Outbreak |
| ORF | 27.4674 | 94.5142 | India | Dhemaji, Assam | (Bora et al., 2016) | Prevalence confirmation |
| ORF | 26.3719 | 91.3821 | India | Nalbari, Assam | (Bora et al., 2016) | Prevalence confirmation |
| ORF | 25.9400 | 119.2300 | China | Farm | (Chi et al., 2013) | Outbreak |
| ORF | 26.1100 | 118.4400 | China | Farm | (Chi et al., 2013) | Outbreak |
| ORF | 26.1000 | 118.5500 | China | Farm | (Chi et al., 2013) | Outbreak |
| ORF | 23.4717 | 77.3908 | India | Bhopal | (Venkatesan et al., 2016) | Case study |
| ORF | 28.4073 | 79.4303 | India | Izatnagar | (Hosamani et al., 2006) | Case study |
| ORF | 27.5985 | 76.5967 | India | Alwar | (Karki et al., 2019) | Case study |
| ORF | 31.2100 | 75.5700 | India | Jalandhar | (Venkatesan et al., 2016) | Case study |
| ORF | 32.1100 | 76.5300 | India | Palampur | (Venkatesan et al., 2016) | Case study |
| ORF | 27.9833 | 79.8324 | India | Shahjahanpur | (Hosamani et al., 2006) | Case study |
| ORF | 27.6015 | 77.6195 | India | Mathura | (Roy et al., 2015) | Case study |
| ORF | 26.2342 | 74.7595 | India | Ajmer | (Venkatesan et al., 2016) | Case study |
| ORF | 26.5200 | 118.8700 | China | Farm | (Chi et al., 2013) | Outbreak |
| ORF | 32.5663 | 46.2785 | Iraq | Sheik Saad | (Al-Bayati, 2022) | Outbreak |
| ORF | 60.1004 | -166.3932 | USA | Nunivak Islands | (Zarnke et al., 1983) | Case study |
| ORF | 63.6299 | -144.6078 | USA | Dry creek | (Zarnke et al., 1983) | Case study |
| ORF | 58.4443 | -134.2341 | USA | Sheep creek | (Zarnke et al., 1983) | Case study |
| ORF | 63.3184 | -143.0754 | USA | Tok | (Zarnke et al., 1983) | Case study |
| ORF | 64.0699 | -145.7123 | USA | Delta Junction | (Zarnke et al., 1983) | Case study |
| ORF | 58.4453 | -134.2330 | USA | Mount Juneau | (Tryland et al., 2018) | Case study |
| ORF | 67.7273 | -164.5387 | USA | Kivalina | (Tryland et al., 2018) | Case study |
| ORF | 57.6500 | -153.6000 | USA | Uyak Bay | (Tryland et al., 2018) | Case study |
| ORF | 71.2733 | -156.7573 | USA | Admiralty Bay | (Tryland et al., 2018) | Case study |
| ORF | 69.3700 | -148.7000 | USA | Sagwon | (Tryland et al., 2018) | Case study |
| ORF | 37.0500 | 37.3700 | Turkey | Gaziantep | (Midilli et al., 2013) | Outbreak |
| ORF | 51.7546 | -1.2409 | England | Oxford | (Gilray et al., 1998) | Outbreak |
| ORF | 53.1000 | -1.5600 | England | Derbyshire | (Gilray et al., 1998) | Outbreak |
| ORF | 56.2440 | -4.3273 | England | Stirlingshire | (Gilray et al., 1998) | Outbreak |
| ORF | 51.4459 | -3.3998 | England | Vale of Glamorgan | (Gilray et al., 1998) | Outbreak |
| ORF | 50.7600 | -3.8100 | England | Devon | (Gilray et al., 1998) | Outbreak |
| ORF | 57.4700 | -4.2200 | England | Inverness | (Gilray et al., 1998) | Outbreak |
| ORF | 54.5900 | -2.8900 | England | Cumbria | (Gilray et al., 1998) | Outbreak |
| ORF | 56.3913 | -3.4475 | England | Perth | (Gilray et al., 1998) | Outbreak |
| ORF | 55.4545 | -4.6194 | England | Ayr | (Gilray et al., 1998) | Outbreak |
| ORF | 50.9460 | -0.1409 | England | Sussex | (Gilray et al., 1998) | Outbreak |
| ORF | 52.1975 | -2.2120 | England | Worcester | (Gilray et al., 1998) | Outbreak |
| ORF | 51.2100 | 0.7300 | England | Kent | (Gilray et al., 1998) | Outbreak |
| ORF | 52.6100 | -1.1200 | England | Leicester | (Gilray et al., 1998) | Outbreak |
| ORF | 52.6200 | -2.7250 | England | Shropshire | (Gilray et al., 1998) | Outbreak |
| ORF | 42.9904 | 20.3346 | Serbia | Tutin | (Milovanovic, 2019) | Outbreak |
| ORF | 44.0262 | 20.4642 | Serbia | Gornji Milanovac | (Milovanovic, 2019) | Outbreak |
| ORF | 5.2768 | 100.5231 | Malaysia | kampung tasek cempedak | (Khoo et al., 2019) | Outbreak |
| ORF | 9.9200 | 8.8900 | Nigeria | Jos-south LGA | (Adedeji et al., 2018) | Outbreak |
| ORF | 12.4700 | 77.0600 | India | Mandya | (Krishnappa et al., 2020) | Outbreak |
| ORF | 39.9856 | -5.2146 | Spain | Oropesa | (Mariscal-Estrada et al., 1992) | Outbreak |
| ORF | 25.9500 | 119.2200 | China | Farm | (Chi et al., 2013) | Outbreak |
| ORF | 26.1000 | 118.6100 | China | Farm | (Chi et al., 2013) | Outbreak |
| ORF | 26.1200 | 118.6000 | China | Farm | (Chi et al., 2013) | Outbreak |
| ORF | 26.0800 | 118.5900 | China | Farm | (Chi et al., 2013) | Outbreak |
| ORF | 26.1600 | 119.0900 | China | Farm | (Chi et al., 2013) | Outbreak |
| ORF | 26.0700 | 119.2100 | China | Farm | (Chi et al., 2013) | Outbreak |
| ORF | 26.0700 | 119.2200 | China | Farm | (Chi et al., 2013) | Outbreak |
| ORF | 26.0800 | 118.5500 | China | Farm | (Chi et al., 2013) | Outbreak |
| ORF | 26.4900 | 118.8900 | China | Farm | (Chi et al., 2013) | Outbreak |
| ORF | 26.4900 | 118.9000 | China | Farm | (Chi et al., 2013) | Outbreak |
| ORF | 25.9200 | 119.3500 | China | Farm | (Chi et al., 2013) | Outbreak |
| ORF | 44.5500 | 124.3100 | China | Farm | (Chi et al., 2013) | Outbreak |
| ORF | 33.4400 | 113.6100 | China | Farm | (Chi et al., 2013) | Outbreak |
| ORF | 25.9500 | 119.2100 | China | Farm | (Chi et al., 2013) | Outbreak |
| ORF | 11.4181 | 76.6476 | India | Ooty | (Balakrishnan et al., 2017) | Outbreak |
| ORF | 37.0866 | 31.7599 | Turkey | Akseki | (Özmen and Dolu, 2018) | Outbreak |
| ORF | 27.5849 | 95.6251 | India | Tinsukia | (Bora et al., 2016) | Prevalence confirmation |
| ORF | 26.7998 | 94.2710 | India | Jorhat | (Bora et al., 2016) | Prevalence confirmation |

**References**

Abrahão, J.S., Borges, I.A., Mazur, C., Lobato, Z.I.P., Ferreira, P.C.P., Bonjardim, C.A., Trindade, G.S., Kroon, E.G., 2012. Looking back: a genetic retrospective study of Brazilian Orf virus isolates. Vet. Rec. 171, 476. https://doi.org/10.1136/vr.100634

Abrahão, J.S., Campos, R.K., Trindade, G.S., Guedes, M.I., Lobato, Z.I., Mazur, C., Ferreira, P.C., Bonjardim, C.A., Kroon, E.G., 2009. Detection and phylogenetic analysis of Orf virus from sheep in Brazil: a case report. Virol. J. 6, 47. https://doi.org/10.1186/1743-422X-6-47

Adedeji, A.J., Adole, J.A., Chima, N.C., Maguda, A.S., Dyek, D.Y., Jambol, A.R., Anefu, E.O., Shallmizhili, J.J., Luka, P.D., 2018. Contagious ecthyma in three flocks of goats in Jos-south LGA, Plateau State, Nigeria. Sokoto J. Vet. Sci. 16, 107–112. https://doi.org/10.4314/sokjvs.v16i1.16

Adedeji, A.J., Maurice, N.A., Wungak, Y.S., Adole, J.A., Chima, N.C., Woma, T.Y., Chukwuedo, A.A., Shamaki, D., 2017. Diagnosis of Orf in west African dwarf goats in Uyo, Akwa Ibom State, Nigeria. Afr. J. Infect. Dis. AJID 11, 90–94. https://doi.org/10.21010/ajid.v11i2.12

Ahanger, S.A., Parveen, R., Nazki, S., Dar, Z., Dar, T., Dar, K.H., Dar, A., Rai, N., Dar, P., 2018. Detection and phylogenetic analysis of Orf virus in Kashmir Himalayas. VirusDisease 29, 405–410. https://doi.org/10.1007/s13337-018-0473-1

Al-Bayati, H.A.M., 2022. Assessment of contagious ecthyma virus in camels of Wasit Province, Iraq. Arch. Razi Inst. 77, 565–571. https://doi.org/10.22092/ARI.2021.356862.1933

Azwai, S.M., Carter, S.D., Woldehiwet, Z., 1995. Immune responses of the camel (Camelus dromedarius) to contagious ecthyma (orf) virus infection. Vet. Microbiol. 47, 119–131. https://doi.org/10.1016/0378-1135(95)00055-F

Baipoledi, E.K., Nyange, J.F.C., Hyera, J.M.K., 2002. A severe case of contagious ecthyma in Tswana goats. J. S. Afr. Vet. Assoc. 73, 86–87. https://doi.org/10.4102/jsava.v73i2.564

Bala, J.A., Balakrishnan, K.N., Abdullah, A.A., Adamu, L., Noorzahari, M.S. bin, May, L.K., Mangga, H.K., Ghazali, M.T., Mohamed, R.B., Haron, A.W., Noordin, M.M., Lila, M.A.M., 2019. An association of Orf virus infection among sheep and goats with herd health programme in Terengganu state, eastern region of the peninsular Malaysia. BMC Vet. Res. 15, 250. https://doi.org/10.1186/s12917-019-1999-1

Balakrishnan, S., Venkataramanan, R., Ramesh, A., Roy, P., 2017. Contagious ecthyma outbreak among goats at Nilgiri hills. Indian J Anim Res 10.

Billinis, C., Mavrogianni, V.S., Spyrou, V., Fthenakis, G.C., 2012. Phylogenetic analysis of strains of Orf virus isolated from two outbreaks of the disease in sheep in Greece. Virol. J. 9, 24. https://doi.org/10.1186/1743-422X-9-24

Bora, M., Bora, D.P., Barman, N.N., Borah, B., Bora, P.L., Talukdar, A., Tamuly, S., 2015. Isolation and molecular characterization of Orf virus from natural outbreaks in goats of Assam. VirusDisease 26, 82–88. https://doi.org/10.1007/s13337-015-0255-y

Bora, M., Bora, D.P., Barman, N.N., Borah, B., Das, S., 2016. Seroprevalence of contagious ecthyma in goats of Assam: An analysis by indirect enzyme-linked immunosorbent assay. Vet. World 9, 1028–1033. https://doi.org/10.14202/vetworld.2016.1028-1033

Castro, E., Pérez, S., Negro, R., Bassetti, L., Rodríguez, S., 2019. Detection and Phylogenetic Analysis of the Orf Virus from sheep in Uruguay. Ann Clin Virol. 1(1): 1002.

Chan, K.-W., Lin, J.-W., Lee, S.-H., Liao, C.-J., Tsai, M.-C., Hsu, W.-L., Wong, M.-L., Shih, H.-C., 2007. Identification and phylogenetic analysis of Orf virus from goats in Taiwan. Virus Genes 35, 705–712. https://doi.org/10.1007/s11262-007-0144-6

Chan, K.-W., Yang, C.-H., Lin, J.-W., Wang, H.-C., Lin, F.-Y., Kuo, S.-T., Wong, M.-L., Hsu, W.-L., 2009. Phylogenetic analysis of parapoxviruses and the C-terminal heterogeneity of viral ATPase proteins. Gene 432, 44–53. https://doi.org/10.1016/j.gene.2008.10.029

Chen, H., Li, W., Kuang, Z., Chen, D., Liao, X., Li, M., Luo, S., Hao, W., 2017. The whole genomic analysis of Orf virus strain HN3/12 isolated from Henan province, central China. BMC Vet. Res. 13, 260. https://doi.org/10.1186/s12917-017-1178-1

Chi, X., Zeng, X., Hao, W., Li, M., Li, W., Huang, X., Wang, S., Luo, S., 2013. Heterogeneity among Orf virus isolates from goats in Fujian province, southern China. Plos one 8, e66958. https://doi.org/10.1371/journal.pone.0066958

Chi, X., Zeng, X., Luo, S., 2017. Diagnosis and phylogenetic analysis of a multifocal cutaneous Orf virus with mixed bacterial infection outbreak in goats in Fujian province, China. Arch. Virol. 162, 2997–3006. https://doi.org/10.1007/s00705-017-3424-z

Coradduzza, E., Sanna, D., Rocchigiani, A.M., Pintus, D., Scarpa, F., Scivoli, R., Bechere, R., Dettori, M.A., Montesu, M.A., Marras, V., Lobrano, R., Ligios, C., Puggioni, G., 2021. Molecular insights into the genetic variability of Orf virus in a Mediterranean region (Sardinia, Italy). Life Basel Switz. 11, 416. https://doi.org/10.3390/life11050416

da Costa, R.A., Cargnelutti, J.F., Schild, C.O., Flores, E.F., Riet-Correa, F., Giannitti, F., 2019. Outbreak of contagious ecthyma caused by Orf virus (Parapoxvirus ovis) in a vaccinated sheep flock in Uruguay. Braz. J. Microbiol. 50, 565–569. https://doi.org/10.1007/s42770-019-00057-7

de Sant’Ana, F.J.F., Leal, F.A.A., Rabelo, R.E., Vulcani, V.A.S., Moreira, C.A., Cargnelutti, J.F., Flores, E.F., 2013. Coinfection by Vaccinia virus and an Orf virus-like parapoxvirus in an outbreak of vesicular disease in dairy cows in midwestern Brazil. J. Vet. Diagn. Investig. Off. Publ. Am. Assoc. Vet. Lab. Diagn. Inc 25, 267–272. https://doi.org/10.1177/1040638713475799

Flores, C., González, E., Verna, A., Peralta, A., Madariaga, C., Odeón, A., Cantón, G., Flores, C., González, E., Verna, A., Peralta, A., Madariaga, C., Odeón, A., Cantón, G., 2017. Virus Orf en humanos, confirmación molecular de un caso clínico en Chile. Rev. Chil. Infectol. 34, 607–609. https://doi.org/10.4067/S0716-10182017000600607

Gelaye, E., Achenbach, J.E., Jenberie, S., Ayelet, G., Belay, A., Yami, M., Loitsch, A., Grabherr, R., Diallo, A., Lamien, C.E., 2016. Molecular characterization of Orf virus from sheep and goats in Ethiopia, 2008–2013. Virol. J. 13, 34. https://doi.org/10.1186/s12985-016-0489-3

Gilray, J.A., Nettleton, P.F., Pow, I., Lewis, C.J., Stephens, S.A., Madeley, J.D., Reid, H.W., 1998. Restriction endonuclease profiles of Orf virus isolates from the British Isles. Vet. Rec. 143, 237–240. https://doi.org/10.1136/vr.143.9.237

Guo, J., Rasmussen, J., Wünschmann, A., de La Concha-Bermejillo, A., 2004. Genetic characterization of Orf viruses isolated from various ruminant species of a zoo. Vet. Microbiol. 99, 81–92. https://doi.org/10.1016/j.vetmic.2003.11.010

Hosamani, M., Bhanuprakash, V., Scagliarini, A., Singh, R.K., 2006. Comparative sequence analysis of major envelope protein gene (B2L) of Indian Orf viruses isolated from sheep and goats. Vet. Microbiol. 116, 317–324. https://doi.org/10.1016/j.vetmic.2006.04.028

Hosamani, M., Yadav, S., Kallesh, D.J., Mondal, B., Bhanuprakash, V., Singh, R.K., 2007. Isolation and characterization of an Indian Orf virus from goats. Zoonoses Public Health 54, 204–208. https://doi.org/10.1111/j.1863-2378.2007.01046.x

Hussain, I., Khan, M., Aslam, A., Rabbani, M., Anjum, A., 2023. Identification, molecular characterization and pathological Features of Orf Virus in sheep and goats in Punjab province, Pakistan. Trop. Anim. Health Prod. 55. https://doi.org/10.1007/s11250-022-03432-z

Inoshima, Y., Ito, M., Ishiguro, N., 2010. Spatial and temporal genetic homogeneity of Orf viruses infecting Japanese serows (*Capricornis crispus*). J. Vet. Med. Sci. 72, 701–707. https://doi.org/10.1292/jvms.09-0467

Inoshima, Y., Murakami, K., Wu, D., Sentsui, H., 2002. Characterization of parapoxviruses circulating among wild Japanese serows (*Capricornis crispus*). Microbiol. Immunol. 46, 583–587. https://doi.org/10.1111/j.1348-0421.2002.tb02738.x

Ito, M., Hayakawa, Y., Shintani, E., Inoshima, Y., 2009. Molecular Characterization of parapoxviruses from infected wild Japanese serows (*Capricornis crispus*) in Ishikawa prefecture (virology). Jpn. J. Zoo Wildl. Med. 14, 103–106. https://doi.org/10.5686/jjzwm.14.103

Karki, M., Kumar, A., Arya, S., Ramakrishnan, M.A., Venkatesan, G., 2019. Poxviral E3L ortholog (viral interferon resistance gene) of Orf viruses of sheep and goats indicates species-specific clustering with heterogeneity among parapoxviruses. Cytokine 120, 15–21. https://doi.org/10.1016/j.cyto.2019.04.001

Karki, M., Kumar, A., Arya, S., Venkatesan, G., 2020. Circulation of Orf viruses containing the NZ7-like vascular endothelial growth factor (VEGF-E) gene type in India. Virus Res. 281, 197908. https://doi.org/10.1016/j.virusres.2020.197908

Khalafalla, A.I., Elhag, A.E., Ishag, H.Z.A., 2020. Field investigation and phylogenetic characterization of Orf virus (ORFV) circulating in small ruminants and pseudocowpoxvirus (PCPV) in dromedary camels of eastern Sudan. Heliyon 6, e03595. https://doi.org/10.1016/j.heliyon.2020.e03595

Khoo, C.K., Norlina, D., Roshaslinda, D., Siti Suraya Hani, M.S., Zuraidah, O., Zunaida, B., Mohd Hasrul, A.H., Roslina, H., 2019. Molecular diagnosis of caprine Orf virus (ORFV) from Penang, Malaysia. Malays. J. Vet. Res. 10, 98–102.

Kinley, G.E., Schmitt, C.W., Stephens-Devalle, J., 2013. A case of contagious ecthyma (Orf Virus) in a nonmanipulated laboratory dorset sheep (*Ovis aries*). Case Rep. Vet. Med. 2013, e210854. https://doi.org/10.1155/2013/210854

Kottaridi, C., Nomikou, K., Teodori, L., Savini, G., Lelli, R., Markoulatos, P., Mangana, O., 2006. Phylogenetic correlation of Greek and Italian Orf virus isolates based on VIR gene. Vet. Microbiol. 116, 310–316. https://doi.org/10.1016/j.vetmic.2006.04.020

Krishnappa, S., Yogisharadhya, R., Roy, P., Bayyappa, M.R.G., 2020. Investigation of mixed infection of goatpox and Orf in Karnataka, India. Authorea Prepr. doi: 10.22541/au.159430864.46044763

Kumar, N., Wadhwa, A., Chaubey, K.K., Singh, S.V., Gupta, S., Sharma, S., Sharma, D.K., Singh, M.K., Mishra, A.K., 2014. Isolation and phylogenetic analysis of an Orf virus from sheep in Makhdoom, India. Virus Genes 48, 312–319. https://doi.org/10.1007/s11262-013-1025-9

Lawal, N., 2020. Molecular characterization and phylogenetic analysis of Orf virus isolated from goats in Sokoto Metropolis, Nigeria. https://doi.org/10.20944/preprints202009.0663.v1

L’Heureux, N., Festa-Bianchet, M., Jorgenson, J.T., 1996. Effects of visible signs of contagious ecthyma on mass and survival of bighorn lambs. J. Wildl. Dis. 32, 286–292. https://doi.org/10.7589/0090-3558-32.2.286

Li, W., Ning, Z., Hao, W., Song, D., Gao, F., Zhao, K., Liao, X., Li, M., Rock, D.L., Luo, S., 2012. Isolation and phylogenetic analysis of Orf virus from the sheep herd outbreak in northeast China. BMC Vet. Res. 8, 229. https://doi.org/10.1186/1746-6148-8-229

Lo, C., Mathisen, G., 1996. Human orf in Los Angeles County. West. J. Med. 164, 77–78.

Lojkic, I., Cac, Z., Beck, A., Bedekovic, T., Cvetnic, Z., Sostaric, B., 2010. Phylogenetic analysis of Croatian Orf viruses isolated from sheep and goats. Virol. J. 7, 314. https://doi.org/10.1186/1743-422X-7-314

Maan, S., Kumar, A., Batra, K., Singh, M., Nanda, T., Ghosh, A., Maan, N.S., 2014. Isolation and molecular characterization of contagious pustular dermatitis virus from Rajasthan, India. VirusDisease 25, 376–380. https://doi.org/10.1007/s13337-014-0205-0

Maganga, G.D., Relmy, A., Bakkali-Kassimi, L., Ngoubangoye, B., Tsoumbou, T., Bouchier, C., N’Dilimabaka, N., Leroy, E.M., Zientara, S., Berthet, N., 2016. Molecular characterization of Orf virus in goats in Gabon, Central Africa. Virol. J. 13, 79. https://doi.org/10.1186/s12985-016-0535-1

Mahmoud, M., Abdelrahman, K., Soliman, H., 2010. Molecular and virological studies on contagious pustular dermatitis isolates from Egyptian sheep and goats. Res. Vet. Sci. 89, 290–294. https://doi.org/10.1016/j.rvsc.2010.02.019

Mansour, K.A., Hussain, M.H., Abid, A.J., Kshash, Q.H., 2021. Orf disease in local goat; clinical and phylogenetic study in Al-Qadisiyah governorate, Iraq. Iraqi J. Vet. Sci. 36, 117–121. https://doi.org/10.33899/ijvs.2021.129489.1651

Mariscal-Estrada, A.L., León-Vizcaíno, L., Cubero-Pablo, M.J., 1992. Investigation of an outbreak of contagious echthyma in the sheep. Av. En Aliment. Mejora Anim. 32, 25–29.

Mazur, C., Ferreira, I.I., Rangel Filho, F.B., Galler, R., 2000. Molecular characterization of Brazilian isolates of Orf virus. Vet. Microbiol. 73, 253–259. https://doi.org/10.1016/S0378-1135(99)00151-0

Midilli, K., Erkılıç, A., Kuşkucu, M., Analay, H., Erkılıç, S., Benzonana, N., Yıldırım, M.S., Mülayim, K., Acar, H., Ergonul, O., 2013. Nosocomial outbreak of disseminated Orf infection in a burn unit, Gaziantep, Turkey, October to December 2012. Eurosurveillance 18. https://doi.org/10.2807/ese.18.11.20425-en

Milovanovic, M., 2019. Detection and phylogenetic analysis of B2L gene of Orf virus from clinical cases of sheep in Serbia. Pak. Vet. J. 39, 433–437. https://doi.org/10.29261/pakvetj/2019.034

Mondal, B., Bera, A.K., Hosamani, M., Tembhurne, P.A. and Bandyopadhyay, S.K., 2006. Detection of Orf virus from an outbreak in goats and its genetic relation with other parapoxviruses. Vet. Res. Commun. 30, 531–539. https://doi.org/10.1007/s11259-006-3270-z

Nagarajan, G., Pourouchottamane, R., Reddy, G.B.M., Yogisharadhya, R., Sumana, K., Rajapandi, S., Murali, G., Thirumaran, S.M.K., Mallick, P.K., Rajendiran, A.S., 2019. Molecular characterization of Orf virus isolates from Kodai hills, Tamil Nadu, India. Vet. World 12, 1022–1027. https://doi.org/10.14202/vetworld.2019.1022-1027

Nóbrega Jr, J.E., Macêdo, J.T.S.A., Araújo, J.A.S., Dantas, A.F.M., Soares, M.P., Riet-Correa, F., 2008. Ectima contagioso em ovinos e caprinos no semi-árido da Paraíba. Pesqui. Veterinária Bras. 28, 135–139.

Oem, J.K., Chung, J.Y., Kim, Y.J., Lee, K.K., Kim, S.H., Jung, B.Y., Hyun, B.H., 2013. Isolation and characterization of Orf viruses from Korean black goats. J. Vet. Sci. 14, 227–230. https://doi.org/10.4142/jvs.2013.14.2.227

Oem, J.K., Roh, I.S., Lee, K.H., Lee, K.K., Kim, H.R., Jean, Y.H., Lee, O.S., 2009. Phylogenetic analysis and characterization of Korean Orf virus from dairy goats: case report. Virol. J. 6, 167. https://doi.org/10.1186/1743-422X-6-167

Olivero, N., Reolon, E., Arbiza, J., Berois, M., 2018. Genetic diversity of Orf virus isolated from sheep in Uruguay. Arch. Virol. 163, 1285–1291. https://doi.org/10.1007/s00705-018-3717-x

Oryan, A., Mosadeghhesari, M., Zibaee, S., Mohammadi, A., 2017. Identification and phylogenetic analysis of contagious ecthyma virus from camels (*Camelus dromedarius*) in Iran. Onderstepoort J. Vet. Res. 84, e1–e5. https://doi.org/10.4102/ojvr.v84i1.1257

Özmen, Ö., Dolu, H., 2018. Pathological and electron microscopical observations on naturally occurring contagious ecthyma outbreak in two wild goats (*Capra aegagrus aegragus*). Ank. Üniversitesi Vet. Fakültesi Derg. 65, 419–423. https://doi.org/10.1501/Vetfak_0000002876

Paiba, G.A., Thomas, D.R., Morgan, K.L., Bennett, M., Salmon, R.L., Chalmers, R., Kench, S.M., Coleman, T.J., Meadows, D., Morgan-Capner, P., Softley, P., Sillis, M., Green, L.E., 1999. Orf (contagious pustular dermatitis) in farmworkers: prevalence and risk factors in three areas of England. Vet. Rec. 145, 7–11. https://doi.org/10.1136/vr.145.1.7

Peralta, A., Robles, C., Martínez, A., Alvarez, L., Valera, A., Calamante, G., König, G.A., 2015. Identification and molecular characterization of Orf virus in Argentina. Virus Genes 50, 381–388. https://doi.org/10.1007/s11262-015-1189-6

Peralta, A., Robles, C.A., Micheluod, J.F., Rossanigo, C.E., Martinez, A., Carosio, A., König, G.A., 2018. Phylogenetic analysis of Orf viruses from five contagious ecthyma outbreaks in Argentinian goats. Front. Vet. Sci. 5. doi: 10.3389/fvets.2018.00134. eCollection 2018.

Roy, R., Tiwari, R., Dutt, T., 2015. Incidence of important goat diseases and economic losses under field condition. Indian J. Anim. Sci. 85(10):1084-1086

Sahu, B.P., Majee, P., Sahoo, A., Nayak, D., 2019. Molecular characterization, comparative and evolutionary analysis of the recent Orf outbreaks among goats in the Eastern part of India (Odisha). Agri Gene 12, 100088. https://doi.org/10.1016/j.aggene.2019.100088

Schmidt, C., Cargnelutti, J.F., Brum, M.C.S., Traesel, C.K., Weiblen, R., Flores, E.F., 2013. Partial sequence analysis of B2L gene of Brazilian Orf viruses from sheep and goats. Vet. Microbiol. 162, 245–253. https://doi.org/10.1016/j.vetmic.2012.10.031

Selim, A., Elhaig, M., Höche, J., Gaede, W., 2016. Molecular detection and analysis of sheeppox and Orf viruses isolated from sheep from Qalubia, Egypt. Berl. Munch. Tierarztl. Wochenschr. 129, 310–317.

Şevik, M., 2017. Association of two clusters of Orf virus isolates in outbreaks of infection in goat in the Central Anatolian region of Turkey. VirusDisease 28, 345–348. https://doi.org/10.1007/s13337-017-0392-6

Sharma, A.K., Venkatesan, G., Mathesh, K., Ram, H., Ramakrishnan, M.A., Pandey, A.B., 2016. Occurrence and identification of contagious ecthyma in blackbuck. VirusDisease 27, 198–202. https://doi.org/10.1007/s13337-016-0316-x

Shehata, A.A., El-Nahas, E.M., Abo Hatab, E.M., Sharawi, S.S.A., Ahmed, H.A., 2021. The genetic identification of camel contagious ecthyma virus as the causative agent of contagious ecthyma in dromedary camels (*Camelus dromedarius*) in Qatar. Trop. Anim. Health Prod. 53, 332. https://doi.org/10.1007/s11250-021-02771-7

Simulundu, E., Mtine, N., Kapalamula, T.F., Kajihara, M., Qiu, Y., Ngoma, J., Zulu, V., Kwenda, G., Chisanga, C., Phiri, I.K., Takada, A., Mweene, A.S., 2017. Genetic characterization of Orf virus associated with an outbreak of severe Orf in goats at a farm in Lusaka, Zambia. Arch. Virol. 162, 2363–2367. https://doi.org/10.1007/s00705-017-3352-y

Suzuki, T., Minamoto, N., Sugiyama, M., Kinjo, T., Suzuki, Y., Sugimura, M., Atoji, Y., 1993. Isolation and antibody prevalence of a parapoxvirus in wild Japanese serows (*Capricornis crispus*). J. Wildl. Dis. 29, 384–389. https://doi.org/10.7589/0090-3558-29.3.384

Tedla, M., Berhan, N., Molla, W., Temesgen, W., Alemu, S., 2018. Molecular identification and investigations of contagious ecthyma (Orf virus) in small ruminants, North west Ethiopia. BMC Vet. Res. 14, 13. https://doi.org/10.1186/s12917-018-1339-x

Tomaselli, M., Dalton, C., Duignan, P.J., Kutz, S., van der Meer, F., Kafle, P., Surujballi, O., Turcotte, C., Checkley, S., 2016. Contagious ecthyma, rangiferine brucellosis, and lungworm infection in a muskox (*Ovibos moschatus*) from the Canadian Arctic, 2014. J. Wildl. Dis. 52, 719–724. https://doi.org/10.7589/2015-12-327

Tomaselli, M., Ytrehus, B., Opriessnig, T., Duignan, P., Dalton, C., van der Meer, F., Kutz, S., Checkley, S., 2022. Contagious ecthyma dermatitis as a portal of entry for *Erysipelothrix rhusiopathiae* in muskoxen (*Ovibos moschatus*) of the Canadian Arctic. J. Wildl. Dis. 58, 228–231. https://doi.org/10.7589/JWD-D-20-00205

Tryland, M., Beckmen, K.B., Burek-Huntington, K.A., Breines, E.M., Klein, J., 2018. Orf virus infection in Alaskan mountain goats, dall’s sheep, muskoxen, caribou and sitka black-tailed deer. Acta Vet. Scand. 60, 12. https://doi.org/10.1186/s13028-018-0366-8

Venkatesan, G., Bhanuprakash, V., Balamurugan, V., Kumar, A., Bora, D.P., Reveniah, Y., Arya, S., Madhavan, A., Muthuchelvan, D., Pandey, A.B., 2016. Simple and rapid visual detection methods of Orf virus by B2L gene based Loop-mediated Isothermal amplification assay. Adv Anim Vet Sci 4, 152–159.

Venkatesan, G., De, A., Arya, S., Kumar, A., Muthuchelvan, D., Debnath, B.Ch., Dutta, T.K., Hemadri, D., Pandey, A.B., 2018. Molecular evidence and phylogenetic analysis of Orf virus isolates from outbreaks in Tripura state of North-East India. VirusDisease 29, 216–220. https://doi.org/10.1007/s13337-018-0442-8

Vikøren, T., Lillehaug, A., Åkerstedt, J., Bretten, T., Haugum, M., Tryland, M., 2008. A severe outbreak of contagious ecthyma (Orf) in a free-ranging musk ox (*Ovibos moschatus*) population in Norway. Vet. Microbiol. 127, 10–20. https://doi.org/10.1016/j.vetmic.2007.07.029

Villadsen, L.S., Zachariae, C.O.C., 2008. Unusual presentation of Orf in an otherwise healthy individual. Acta Derm. Venereol. 88, 277–278. https://doi.org/10.2340/00015555-0399

Wang, Y., Yang, K., Wang, Y.A., Yu, Z., Zhang, Q., Zhang, G., Su, L., Lu, Z., Zhang, X., Liu, Z., Jiang, S., Li, Y., 2019. Identification and phylogenetic analysis of an Orf virus strain isolated in Anhui Province, East-central China, in 2018. Acta Virol. 63, 270–277. https://doi.org/10.4149/av_2019_304

Yogisharadhya, R., Bhanuprakash, V., Venkatesan, G., Balamurugan, V., Pandey, A.B., Shivachandra, S.B., 2012. Comparative sequence analysis of poxvirus A32 gene encoded ATPase protein and carboxyl terminal heterogeneity of Indian Orf viruses. Vet. Microbiol. 156, 72–80. https://doi.org/10.1016/j.vetmic.2011.10.021

Zarnke, R.L., Dieterich, R.A., Neiland, K.A., Ranglack, G., 1983. Serologic and experimental investigations of contagious ecthyma in Alaska. J. Wildl. Dis. 19, 170–174. https://doi.org/10.7589/0090-3558-19.3.170

Zhang, K., Lu, Z., Shang, Y., Zheng, H., Jin, Y., He, J., Liu, X., 2010. Diagnosis and phylogenetic analysis of Orf virus from goats in China: a case report. Virol. J. 7, 78. https://doi.org/10.1186/1743-422X-7-78

Zhang, K., Xiao, Y., Yu, M., Liu, J., Wang, Q., Tao, P., Liu, S., Ning, Z., 2015. Phylogenetic analysis of three Orf virus strains isolated from different districts in Shandong Province, East China. J. Vet. Med. Sci. 77, 1639–1645. https://doi.org/10.1292/jvms.15-0368
