# Supplementary material for: An evaluation of the ecological niche of Orf virus (Poxviridae): Challenges of distinguishing broad niches from no niches
Source: PLoS One. 2024 Jan 18;19(1):e0293312. doi: 10.1371/journal.pone.0293312 (PMC10796068; doi:10.1371/journal.pone.0293312)
Supplement: S8 Table — (DOCX) [file pone.0293312.s009.docx]

| **Features** | **Maxent** | **NicheA** |
| --- | --- | --- |
| Availability | Free and open source | Free and open source |
| Visualization of Hutchinsonian duality | No | Yes |
| Model visualization in G | Yes | Yes |
| Model visualization in E | No | Yes |
| L1-Regularization | Yes | No |
| Different levels of constraints (linear, quadratic, product, threshold, hinge, and categorical) | Yes | No |
| Quantification of niche overlap | No | Yes |
| Creating virtual species | No | Yes |
| Creating hypothesized geographic barriers | No | Yes |

G – Geographic space, E- Environmental space
