# Supplementary material for: An evaluation of the ecological niche of Orf virus (Poxviridae): Challenges of distinguishing broad niches from no niches
Source: PLoS One. 2024 Jan 18;19(1):e0293312. doi: 10.1371/journal.pone.0293312 (PMC10796068; doi:10.1371/journal.pone.0293312)
Supplement: Attachment — Submitted filename: Response to reviewers.docx [file pone.0293312.s010.docx]

**Response to Reviewers’ Comments**

**Reviewer #1**

- In this manuscript, the authors analyse the ecological niche of the Orf virus, a cosmopolitan and obligate pathogen of ruminants, in an attempt to discern its limits. I really appreciate the modelling effort and meticulousness applied in this study. However, I have some concerns about a few methodological decisions that might had obscure the results. The failure to identify the niche limits might be related to two issues: i) a slight temporal discrepancy between outbreaks and the environmental data used; and ii) the use of principal components instead of a selection of the original environmental variables. Before acceptance, I strongly encourage the authors to consider these two issues in order to strengthen their conclusions.
- We really appreciate the reviewer’s encouraging remarks on the manuscript. We would like to express our gratitude to the reviewer for having spent considerable time to review this manuscript. We believe that we have taken adequate care in responding to all of the suggestions by the reviewer… our responses to each of the reviewer’s comments are below.

i) a slight temporal discrepancy between outbreaks and the environmental data used:

*We did not want to restrict the records to those that were reported before the year 2000 due to the following reasons:*

- *Recent increase in reports: We learnt from our literature survey that the factors such as presence of veterinary colleges and hospitals, diagnostic facilities in such institutions, and/or access to farms for veterinarians etc., greatly influence the reporting of Orf prevalence in developing countries. Because of the recent development of infrastructure and diagnostic facilities in developing countries, last decades witnessed reporting of many Orf cases. This is one of the major reasons in the inclusion of many cases from recent decades.*
- *Continuous presence in environment and high resilience to environmental degradation; hardy in nature. Orf virus is very resistant to climatic conditions (hardy in nature (Bergqvist et al., 2017)), and can survive in environment for many years. Spyrou and Valiakos (2015) reported that Orf virus can be viable in environment for up to 17 years, and Orf virus has high resilience to environmental degradation (Hsu et al., 2016). These viruses exhibit high ability to survive in harsh environmental conditions (Bukar et al., 2021; Al Saad et al., 2017). Continuous presence of Orf virus in environment was reported to be one of the major reasons behind re-emergence of Orf virus infections (Kumar et al. 2015; Kumar et al 2022).*
- *Many routes of infection from inanimate objects: Frequent animal movement from one geographical location to another, and capability of Orf virus to remain viable in farm buildings, fomites, farm equipment, and environments for long term (months to years) increases the risk of Orf infections.*

*Considering all of the above factors, two in particular: continuous presence of Orf virus in environment and high resilience to environmental degradation, we did not want to restrict the occurrence points to those that were reported before the year 2000. Rather, we opted to include as many and as diverse occurrence data as possible, such that our occurrence data were more broadly representative of the environments under which the virus is found.*

*References*

1. *Doi: 10.1016/j.vetmic.2015.08.010*
2. *Doi: 10.3390/vaccines9111341*
3. *Doi: 10.9790/2380-1007016469*
4. *Doi: 10.14737/journal.aavs/2015/3.12.649.676*
5. *Doi: 10.1007/s11250-022-03269*
6. *Doi: 10.1093/infdis/jiw307*
7. *Doi :10.1002/rmv.1932*

*In the 4^th^ paragraph of the Methods section, we have added a sentence to explain these points and make clear the reasons behind our methodological decisions.*

ii) the use of principal components instead of a selection of the original environmental variables

- *Our response to this point is explained below in the same reviewer’s comments about our Methods section.*

Introduction

- 1st paragraph, line 1: The terms “contagious pustular dermatitis”, “sore mouth” and “contagious ecthyma” are terms referring to the same condition? If it is so, it should be properly stated. For instance, “Contagious pustular dermatitis, sore mouth or contagious ecthyma (CE) are the given names of a viral skin disease…”
- *As suggested by the reviewer, the sentence has been modified for greater clarity.*
- 1st paragraph, line 3: Please avoid acronyms and abbreviations at the beginning of the sentence (check in the entire text).
- *As suggested, ‘CE’ is expanded as “Contagious ecthyma”.*
- 1st paragraph, lines 10-12: Divide in two independent sentences. “…on the dorsal aspect of the hands [5]. Infection occurs less…”.
- *As suggested, the long sentence is now divided into two independent sentences.*
- 1st paragraph, lines 12-13: Remove colon after “CE infections [4]”. What references the citation [4]? The self-limiting nature? In such a case, the citation should be located with the proper piece of information.
- *Citation [4] is for self-limiting nature. As suggested, the reference is now properly placed.*
- 2nd paragraph, lines 8-10: “…from THE environment…”
- *Done.*
- 3rd paragraph: Has a minor contribution to introduce the subject, and can be removed.
- *As suggested, the 3^rd^ paragraph is deleted.*

Materials and Methods

- 1st paragraph, lines 3-4: the search terms should not be abbreviated or replaced by acronyms.
- *The search terms (abbreviated and non-abbreviated), mentioned in the materials and methods were used as such for the search, as some of the research is indeed listed under abbreviated names.*
- 2nd paragraph, line 7: “…determination of the centroid of that ADMINISTRATIVE area, calculation of its geographic EXTENSION…”.
- *The sentence is modified as suggested.*
- 3rd paragraph, line 3: Replace [40,39,41] with [39-41].
- *The reference format is changed as suggested.*
- 4th paragraph, lines 1-2: The WorldClim 2.1 database is a climatic consensus comprising the period 1970-2000, whereas your data comprises from 1971-2021. How did the authors deal with this temporal discrepancy?
- *Response to this comment is provided above, under the responses to the reviewer’s general comments.*
- 4th paragraph, lines 5-7: Considering PCA to reduce dimensionality of the environmental variables is an appropriate approach. However, given the results obtained, have the authors consider the possibility of selecting a number of sets of the original variables and repeat their modelling experiments? I know it represents a great deal of work, but the results that could be obtained may reinforce (or not) the conclusions of the present study.
- *Thanks for this suggestion. We note that all models do one of two things: they can explain, or they can predict. A model designed to explain is one where we would wish to retain the original variables, as such a model would be maximally interpretable. In this case, however, our goal was to predict suitability across landscapes/regions, and so interpretability of independent (environmental) variables is less necessary. Rather, we would wish to retain a maximum of information in those variables, rather than emphasizing their interpretability. We note that if we could not “predict” using the prediction-optimized PCA variables and prediction-focused modeling approaches, any association with individual (raw) variables is likely to be spurious.*
- *In this context, we are aiming to use the most climatic information available to us in our analyses. Correlation-based variable removal focuses on pairs of variables correlated at, say r > 0.8 or 0.9… although those pairs of variables mostly covary, they do have some independent variation, and potentially some independent information content, which is lost if we eliminate variables. The PCA, on the other hand, in theory retains that information in new axes that, while less interpretable, retain the full original information. More information about the utility of PCA and our methodological choices has been added to the Methods.*

5th paragraph, lines 5-6: Were every combination between the five principal components considered? Or only the set comprising the principal components 1 to 5?

- *Here, we were talking about the very early modeling experiment, and in that experiment, we used a set comprising the first five PCs. No combinations were used. We entered into considerably greater depth in subsequent modeling efforts, detailed later in the Methods.*
- 5th paragraph: How were these models assessed?
- *Method of assessing model performance is mentioned in the methodology section:* “*Final models were selected from among candidate models based on three criteria: statistical significance, omission rate, and lowest Akaike information criterion (AICc) values, as suggested by Cobos et al. [52]. Statistical significance of models was tested using partial receiver operating characteristic (pROC) statistics [54]. Statistically significant models were filtered using omission rate, retaining only those models for which omission rate was <5% [55]. Statistically significant models with <5% omission rate were ranked based on AICc values and the models that are within 2 AICc units of the minimum were chosen as final models. …….. From the set of final models, the best model was identified as the one with a delta_AICc value of zero (i.e., difference in AIC value between the top model and the remaining models in the final set)*.”
- 6th paragraph, lines 1-3: Please explain how these 20 models were selected.
- *See preceding response. In each of the 20 experiments, the above method was followed*.
- 7th paragraph: Please specify the axes (and variables) selected to construct and represent the minimum volume ellipsoids.
- *It is mentioned that first three PCs were used to construct minimum volume ellipsoid.* “*Seventy percent of replicate occurrence data and first three PCs (PC1, PC2, and PC3) were used to implement the MVE*”

Results

- 1st paragraph, line 2-3: How many candidate models were assessed?
- *Sixty candidate models were assessed. The sentence has been modified to make this point more clearly.*
- 1st paragraph, lines 4-5: Relying the calculation of the AUC on a presence/absence confusion matrix (and thus in true absences), why did the authors use this discrimination metric instead of a more appropriate one, such as the partial ROC? (Peterson et al 2008, doi: 10.1016/j.ecolmodel.2007.11.008).
- *Please note that we did not use traditional AUC to assess the model performance, and we used partial ROC tests to choose statistically significant models. It is mentioned in the manuscript:* “*Statistical significance of models was tested using partial receiver operating characteristic (pROC) statistics” To avoid confusion, the key sentence is modified in the results as follows: “Statistically significant partial AUC ratios (mean ratio 1.116 ± 0.002, all P < 0.001) suggested that the predictions of those models were better than random predictions”*
- *To further avoid confusion, we have incorporated the following lines in the methodology. “In traditional AUC approach, commission and omission errors are given equal weightage in assessing the model performance [59]. As we used only presence-only data, partial AUC ratio, determined using partial ROC method was employed to analyze the model performance [56]. In this method, x - axis of traditional AUC-ROC (commission error) is replaced with proportion of the whole study area predicted as present to restrict the AUC calculations within the regions where model predicts [56], and under the restricted curve, only the relevant regions that meet the user-defined omission threshold (E = 5%) were considered. Partial AUC ratio is defined as the ratio of AUC of the restricted ROC curve to the AUC of the restricted random expectation curve [56]. Partial AUC ratio values range from 0 to 2, and a value of 1 indicates random performance [56,60]. Partial AUC ratios were averaged across 10 replicates of final models, and represented as mean partial AUC ratios”*
- 2nd paragraph, lines 1-4: How many models were finally assessed? And how many were selected in each of the partition experiments?
- *A separate supplementary file is prepared (Suppl. file 7), and is cited in the text of the Results; all information related to total number of assessed and selected models were provided.*
- Figure 4: What does each of the 4 maps indicate? This figure panel should be properly arranged, with each of the maps being individually identified and explained in the figure legend. Furthermore, their interpretation will benefit of superimposing the original occurrence data (3rd paragraph, lines 1-4).
- *Figure 4 indicates a set of representative models. We are not giving separate description to each of these models as our emphasis is to show consistent and repeated omission of known distributional areas in the predictions. As suggested by the reviewer, occurrence points (white circles) were plotted on each of the models.*

Figure 5: The interpretation of this figure will also benefit of superimposing the original occurrence data (3rd paragraph, lines 4-6).

- *As suggested by the reviewer, the occurrence points were plotted on the map.*

3rd paragraph, lines 6-11: It is unclear how many models met the omission rate criteria in each of the partition sets. I guess there is one model out of 20 in the three-partition models and none in the two-partition models. Please check and rephrase.

- *As suggested by the reviewer, the sentence is modified as follows: “Evaluating the selected models using two partly independent test datasets (IND1 and IND2) sourced from the occurrence pool (i.e., three-partition models) revealed that only one model out of 10 final models met our omission rate criteria (<5%). No model met our omission rate criteria among the 10 models based on two-partition sets. All final models in both two- and three- partition experiments were statistically significant (P < 0.001, Tables 1 and 2)”*

3rd paragraph, lines 11-14: Where are these results presented?

- *We mentioned a part of our observation in results (3^rd^ paragraph, lines 1-4), and also in the discussion (3^rd^, 4^th^, and 5^th^ paragraphs). For clarity, the sentence is modified as follows: “Results of independent final evaluation of selected models thus coincided with manual assessment of predicted models based on distributional knowledge (see discussion for more details): our models failed to predict distributional potential of Orf virus in several areas across the world”*

4th paragraph, lines 1-3: Which one of the Maxent models? How was it determined as representative? I dare guess it is irrelevant which one of them. If it is so, you should rephrase as “…used to calibrate Maxent models…”.

- *Thanks for this suggestion. As suggested, the sentence is rephrased.*

Figure 6: This figure panel must be improved, as visualization is less than optimal (especially in the top-left figure). Moreover, axis titles and appropriate references are missing.

- *As suggested, figure panel is improved and figure legends are modified for better understanding.*

4th paragraph: Each of the mentions to Figure 6 should specify to which panel section is referring to.

- *As suggested, figure panels are properly mentioned in the text.*

It is particularly surprising that, given that the authors have decided to consider the location uncertainty of the outbreaks, the suitability maps provided are not accompanied by the corresponding uncertainty maps. To inform the uncertainty of the modelling procedures is not only strongly recommended, but provides a valuable aid in the interpretation of the results.

- *As a supplementary figure (8), cited in the text, uncertainty maps are provided. Method of estimating uncertainty is mentioned in the methodology (last sentence of 6^th^ paragraph of methodology), and our observations are mentioned in the results (last sentence of 3^rd^ paragraph of results).*

Discussion
1st paragraph: Rephrase “In spatial epidemiology, THE lack of knowledge regarding the precise location of outbreaks, and possibilities of hosts, AND moving away from the exact location prior to the initial documentation of infection, can affect the model quality by reducing the effective sample size…”

- *The sentence has been rephrased.*

2nd paragraph, line 3: Remove “statistically”.

- *It is removed.*

3rd paragraph: How can the authors be confident enough to suspect of their models based on those doubtful outbreaks of Orf virus in Russia? If they rely on that data, none of those outbreaks could be included in the modelling procedures?

- *From the given references [71 - 77], it was clear to us that Orf prevalence was noticed in Russia. However, we were not able to get reliable administrative areas in Russia to convert such information into occurrence records by assigning uncertainty.*

8th paragraph, line 2: Remove “statistically”.

- *It is removed*

**Reviewer #2:**

- Consider citing 10.1007/s11250-022-03269-6.
- *Thanks for this suggestion. We have cited the paper.*
- Please consider biased reporting of cases especially in developing countries like India, where there are reports from specific locations where there are facilities like veterinary colleges and research institutes.
- *We really appreciate this comment. We understand the biasedness in reporting in developing countries owing to the availability / non-availability of facilities and infrastructure. As our focus was to develop the global ecological niche of Orf virus, we have used the available records. We have indicated in the last paragraph of the discussion that availability of large sample size may help to improve the niche identification issues related to Orf virus.*
