# Supplementary material for: An evaluation of the ecological niche of Orf virus (Poxviridae): Challenges of distinguishing broad niches from no niches
Source: PLoS One. 2024 Jan 18;19(1):e0293312. doi: 10.1371/journal.pone.0293312 (PMC10796068; doi:10.1371/journal.pone.0293312)
Supplement: Attachment — Submitted filename: Response to Reviewers.docx [file pone.0293312.s011.docx]

**Response to Reviewers’ Comments**

**Reviewer #1**

- I am glad to see that most of my comments have been fully considered, and that the manuscript has notably improved. Despite a minor recommendation below, as far as I am concerned, this manuscript is now suitable for its publication.
- *We express our deep gratitude to the reviewer for helping us to improve the manuscript considerably.*
- Concerning the justification for solely using PCA components and not the original environmental variables (previously commented in Materials & Methods, 4th paragraph), and since the issue might be a common concern among readers, I believe this must be included in the Discussion.
- *Thanks for this suggestion. As suggested by the reviewer, we incorporated the details related to PCA in the first paragraph of the Discussion.*

**Reviewer #3:**

- To date, there are many scientific works in the field of virology based only on bioinformatics data (in silico). Unfortunately, there is very little confirmation of all bioinformatic data by field studies.
- *We appreciate the reviewer’s statement, but we are not entirely sure of their point. In our paper, we are trying to take results from detailed laboratory studies, and interpret them in terms of the suitability of landscapes/regions for Orf virus across the world by using an ecological niche modeling approach.*
- *In the abstract, we have mentioned clearly the challenges involved in interpreting our results as follows: “These results suggest two possibilities: that the niche models fail to identify niche limits that constrain the virus, or that the virus has no detectable niche, as it can be found throughout the geographic distributions of its hosts.”*
- *In the manuscript, we suggested the importance of cross-verifying the model predictions with existing field-based distributional knowledge in several places. In the Results: “However, manual assessment of models (Fig 4) based on the available distributional knowledge of Orf virus revealed that all of our models failed consistently to predict known occurrences in many parts of the world…” and “Results of independent final evaluation of selected models thus coincided with manual assessment of predicted models based on distributional knowledge (see Discussion for more details): our models failed to predict distributional potential of Orf virus in several areas across the world.”*
- *In the Discussion is this text: “We sought to answer the niche question for Orf virus via two assessments: (1) Do generated models show non-random patterns of model failure? (2) Can we see consistent and repeated patterns of omission in the models generated? In this latter step, we assessed the models developed by integrating additional distributional knowledge, and noticed that significant parts of the known distribution of Orf virus were not represented in the geographic space visualizations.”*
- *More generally, we point to the title of our manuscript, indicating that there are challenges associated with identifying broad niches from no niches in the case of globally distributed pathogens. We believe that this study will help researchers to acknowledge certain potential caveats in interpreting model results realistically. In this sense, we believe that we are in agreement with the reviewer’s comment.*
- The use of the technique "Peterson, Samy, 2016" needs to be explained and shown to be possible for Poxviridae. The authors (Peterson, Samy, 2016) use it for completely different viruses with completely different ways of spreading in nature.
- *Peterson and Samy (2016) used buffer zones of varying sizes around each occurrence record to address uncertainty in model outputs. The method employed is not species-specific, which is to say that, if uncertainty exists in the spatial localization of species occurrence records, this method helps researchers to incorporate the uncertainty directly into the analysis process. This method was particularly suitable in our case as we did not have very precise geographical coordinates for many records.*
- The authors state "thus enhances our ability to deal with varying degrees of uncertainty associated with precise localization." Is there at least one confirmation of this postulate in the scientific literature? If so, why is it not cited? Or is it just unconfirmed thoughts of the authors?
  - *The preceding part of the sentence had the reference that the reviewer is seeking. However, to avoid confusion, we have now placed this reference at the end of the sentence.*
- In the study, the authors use the "NicheA" platform used for niche visualization in environmental space for a specific virus - Orf Virus. To confirm the capabilities of this technique, several literary sources are used (numbers 50-54). However, these articles do not use viruses as a model; moreover, they were published several years earlier than the proposed methodology. How can the proposed methodology be justified in this way?
- *Sorry for the confusion. The references to which the reviewer refers were not used for supporting the capabilities of NicheA. Rather, those references were key in supporting underlying assumption, such as the convex nature of species’ fundamental niches, which is the key idea in the NicheA algorithm.*
- *Considering the reviewer’s suggestion to incorporate more works related to niche modeling studies of viruses in which NicheA was employed, we have included additional references (117, 118 & 119) in line 3 of paragraph 7 in discussion.*

- Despite the unsuccessful predictions of previous models, the authors argue that there is an opportunity to improve the ability to predict epidemics, but their description and comparison requires more meaningful evaluation data.
  - *We have mentioned in the article that the ENM approaches have limitations in developing realistic models of globally distributed viruses: “These results suggest two possibilities: that the niche models fail to identify niche limits that constrain the virus, or that the virus has no detectable niche, as it can be found throughout the geographic distributions of its hosts.” (see abstract, lines 18-20)*
  - *Considering the potential limitations and inferior quality of models developed, we concluded in the discussion that “However, confirming whether or not Orf virus has a detectable niche separate from that of its host remains a challenge, awaiting much larger sample sizes, and information on host-breadth [35] (i.e., accessible host populations for Orf virus to infect and survive in the absence of dispersal barriers). Host-breadth identification may reveal relevant biotic factors that can inform component-based ENM of pathogens [35].*
  - *As such, in this article, our focus was to assess the models’ capability to predict, and we noted that models failed to predict suitability in several known-distributional areas across the world. We believe that this study will help the researchers to acknowledge certain potential caveats in realistically interpreting model results. As such, we believe that our Discussion of our results is actually in agreement with the reviewer’s comments.*
- The NicheA program is practically not used for analysis in virology, therefore, additional explanations for the choice of this software are required.
- *NicheA has indeed been used for assessing the ecological niches of viruses in environmental space, previously, as can be appreciated from the citations listed above. More broadly, though, NicheA is just a tool for visualization and analysis of data, and is not the focus of this paper. In this paper, we marshall several novel tools for this analysis … but the point of the paper is to assess whether a detectable ecological niche exists for a globally distributed virus.*
- How correct is it to cite an article (Low et al 2020) in the legend for Figure 5?
- *We do not think that it is uncommon or inappropriate to cite references in the figure legends, but the editors of the journal can decide whether that is something to be avoided.*
- The authors compare different methods of virus ecology analysis. For a better perception, such an analysis should be accompanied by a table with comparisons of various programs.
  - *We have added such a table, in the supplementary materials.*
